# Methylation Changes of Primary Tumors, Monolayer, and Spheroid Tissue Culture Environments in Malignant Melanoma and Breast Carcinoma

**DOI:** 10.1155/2019/1407167

**Published:** 2019-01-17

**Authors:** Özge Şükrüoğlu Erdoğan, Seda Kılıç Erciyas, Ayhan Bilir, Şeref Buğra Tunçer, Demet Akdeniz Ödemiş, Sıdıka Kurul, Hasan Karanlık, Neslihan Cabıoğlu, Hülya Yazıcı

**Affiliations:** ^1^Istanbul University, Institute of Oncology, Department of Basic Oncology, Cancer Genetics Division, Istanbul, Turkey; ^2^Istanbul Aydin University, Faculty of Medicine, Department of Histology and Embryology, Istanbul, Turkey; ^3^Istanbul University, Institute of Oncology, Department of Clinical Oncology, Surgical Unit, Istanbul, Turkey; ^4^Istanbul University, Istanbul Faculty of Medicine, Department of Surgery, Istanbul, Turkey

## Abstract

Epigenetic changes have major role in the normal development and programming of gene expression. Aberrant methylation results in carcinogenesis. The primary objective of our study is to determine whether primary tumor tissue and cultured tumor cells in 2D and 3D tissue culture systems have the same methylation signature for* PAX5*,* TMPRSS2*, and* SBDS*. These findings will play an important role in developing in vitro model system to understand the effect of methylation inhibitors on primary tumor tissue. In a previous study* PAX5*,* TMPRSS2*, and* SBDS* genes that we are investigating were reported to be methylated more than 60% in breast cancer and malignant melanoma cell lines. However, these genes have never been studied in primary tumor tissues. Thus, primary tumor tissues of breast cancer and malignant melanoma were first grown in 2D and 3D cultures. Then these two types of tumor tissues and their 2D and 3D cultures were investigated for changes considering methylation levels in* PAX5*,* TMPRSS2*, and* SBDS* genes using real-time polymerase chain reaction. No differences were observed in the primary tissues and culture systems for both* PAX5* and* TMPRSS2* in malignant melanoma tissues. We found that* PAX5* gene was an efficient marker to measure the effects of methylation inhibitors for in vitro systems for malignant melanoma tissue.

## 1. Introduction

Malignant melanoma is a carcinoma of the skin that grows as a result of malignant transformation of melanocytes in the basal layer of the epidermis and it is the most aggressive of all skin cancers [1]. Cancerous cells develop due to the deterioration in skin cells damaged through UV radiation and sunshine or due to damage in DNA. Five percent of all skin carcinomas have been reported as malignant melanoma but it is the type that is most responsible for skin cancer deaths [2]. Recent studies showed that DNA methylation was associated with proliferative and immunologic processes in subgroups of melanoma [3–5]. Recently tumor suppressor genes (TSGs) have been shown to play a role in the pathogenesis of various cancer types. More than 100 genes contributing to the pathogenesis of malignant melanoma have been found to be abnormally hypermethylated. Abnormal methylation of TSGs is thought to be associated with aggressive clinicopathological features and poorer survival. Epigenetic changes may be a significant prognostic marker that benefits routine practice [6]. In general, the DNA methylation profile of cancer cells is characterized by global genomic hypomethylation accompanied by specific hypermethylation on tumor suppressor genes, resulting in metastasis and poor clinical outcomes [7].

Breast cancer develops as a result of the uncontrolled proliferation of cancer cells in breast tissue. The incidence of this disease is increasing worldwide. Germline mutations of breast cancer susceptibility genes are responsible for hereditary breast cancers [8]. Also, mutations and epigenetic changes affect the development of breast cancer [9]. In the analysis of whole genome gene expression microarray and DNA methylation microarray, genes with abnormal DNA methylation were investigated for early detection in breast cancer. Recent studies have shown that changes in DNA methylation at an early stage in the development of breast cancer (BC) may be clinically appropriate for therapeutic decisions [10].

To date, at least 50 different genes have been demonstrated to have the rearranging function of DNA methylation and histone modifications in malignant melanoma [11]. In the study by Echrich et al. (2008), a high rate of methylation was shown in* PAX5*,* TMPRSS2*, and* SBDS* genes of various cell lines. The authors found non-similar methylation patterns of breast cancer samples although strong cluster formation was observed for colon cancer, CNS, and melanoma when the relationship was analyzed between the cell lines [12].* PAX5* gene is basically involved in the development of B cells. In the absence of this gene, these cells can turn into T cells or natural killer cells [13].* PAX5* was found as a potential immunohistochemical marker in differential diagnosis of lymphoid neoplasms [14].* TMPRSS2 *is included in the family of transmembrane serine protease type 2 genes and was found in the basal cells of prostate epithelium [15]. Although the exact function of gene is unknown, it was determined that it modulates sodium current in the epithelial sodium channel of lungs [16]. Although the function of* SBDS *is not fully understood, some studies have shown that it is involved in the maturation and processing of RNA and ribosomes [17, 18].

Methylation inhibitors are not used in solid tumors because of their unknown adverse effects and its usage is only to hematologic malignancies [19]. Methylation inhibitors such as 5-azacytidin and 5-azadeoxycytidin reversibly inhibit DNA methyltransferases and thus, hypomethylation of DNA has occurred [4]. This activity may activate tumor suppressor genes that have the antitumor effect silenced by hypermethylation and oncogenes have oncogenic effect by hypermethylation. As a consequence,* in vitro* model systems are required in patients especially with solid tumors for measuring the adverse effects of methylation inhibitors. Hence, it is very important to measure antitumor effects of methylation inhibitors before using as chemotherapeutic agents for patients with solid tumors.

In the study by Harman et al. (2016), the effects of the DNA methyltransferase (DNMT) inhibitors such as 5-azacytidine (5-AzaC) were examined on normal and tumoral mammary cell lines derived from dogs, cats, and humans whether they were useful models for human breast cancer. The study showed that the effects of 5-AzaC varied on gene expression between the different species and different tumor cell lines of the same species. The results of study were confirmed in primary malignant cells isolated from dog and cat adenocarcinomas. These findings suggest that three species, dogs, cats, and human, may be useful models for the preclinical evaluation of new therapeutics in human breast cancer studies [20].

Nowadays, it is known that 3D tumor tissue culture systems have 85% similarity with primary tumor tissue. Therefore, 3D tumor tissue culture systems can be used as* in vitro* model systems for these purposes. But, specific genes are needed to know as a biomarker for each specific tissue. These genes have to show the same features of methylation in tumor cell grown in 2D and/or 3D culture systems compared with primary tumor tissue.

The main goal of the study is to discover an* in vitro* model system and associated tools for measuring the effects of methylation inhibitors. For this reason, the primary objectives were to grow tumor cells in 2D and 3D tissue culture systems and to determine whether the cells grown in the culture systems and primary tumor cells had the same features of methylation in the genes* PAX5, TMPRSS2, and SBDS*.

In this study, the genes were selected from the cell line of associated tissues that have previously reported in literature (Ehrich, Turner et al. 2008). These genes were found to be hypermethylated in breast cancer and malignant melanoma cell lines. However, the methylation status of selected* PAX5, TMPRSS2,* and* SBDS* genes was examined in cell lines but not primary tumor tissue of patients with breast cancer and malignant melanoma in the literature. For this reason, these genes were selected to determine the methylation levels in primary tumor tissue and to find out the difference between the primary tumor tissue and the 2- and 3-dimensional cell cultures.

One of the most effective way to display tumor tissue changes is 3D culture systems. If the changes in 3D cultures are the same with primary tumor tissues,* in vitro* 2D and/or 3D tissue culture systems could be used as a model to detect methylation changes. In this way, the expression levels of genes converted to unmethylated levels with methylation inhibitors could be followed* in vitro*. Moreover, the interactions between cells and the interested gene could be understood with* in vitro* 3D tissue cultures.* In vitro* 3D culture systems could be used to estimate the benefits of methylation inhibitors in the treatment of tumors and also to predict the adverse effects of methylation inhibitors in patients.

## 2. Materials and Methods

### 2.1. Patient Groups and Preparation of Specimens

Primary tumor tissues were obtained from 10 patients with breast cancer and from 6 patients with malignant melanoma with a presumed diagnosis of cancer which were admitted to the Surgical Unit of Clinical Oncology Department in Oncology Institute at Istanbul University. The study was approved by the Ethics Committee of Istanbul Medical Faculty at Istanbul University (2009/2524-7).

Two pieces of 0.5 cm^3^ Tru-cut tissue biopsies were extracted from primary tumors during surgery for the analysis. Under sterile conditions, one of the tissue biopsies was placed in 10 mL RPMI 1640 solution including 10% phosphate-buffered saline (PBS) for tissue culture, and the other tissue biopsy was treated with RNAlater solution (Qiagen Inc.) for DNA/RNA studies. The tissue piece in the RNAlater was stored for 1 night at 4°C and then transferred to a liquid nitrogen cryo-resistant tube and stored in a liquid nitrogen tank. The tissue piece in the RPMI 1640 (Biochrom) was shredded into pieces for primary tissue culture.

### 2.2. Preparation of Primary Cell Cultures

In a sterile laminar flow, the shredded tissue was planted in a 25 cm^2^ cell culture flask by adding 5 mL of DMEM-F12 medium (Biochrom). Flasks were placed into an incubator containing 5% CO_2_ at 37°C. Flasks were left to culture for 5 days without being moved. At the end of the 5th day, flasks were examined with an inverted microscope to observe the cellular proliferation. The media in the tissue culture flask were first replaced on the 5th day and refreshed every 2 days thereafter. When flasks became confluent, 3D tissue culture processes were started. All 2D and 3D cultures were planted into 3 flasks against contamination risk and coefficient variations between flasks.

### 2.3. Cultivation of Three-Dimensional Spheroid Model

The confluent flasks were trypsinized with 0.05% trypsin. Half of the cells were used for spheroid cultivation; the other half were stored for DNA/RNA/protein isolation from the monolayer culture. The liquid overlay method was used for manufacturing the multicellular tumor spheroids. 3% Agar stock solution was prepared. A six-well culture plates were coated with 1/1 ratio agar/medium mixture. Flasks were incubated for 3 days in the incubator containing 5% CO_2_ at 37°C. After 3 days, spheroids were harvested and DNA/RNA/protein isolation was performed.

### 2.4. Concurrent DNA/RNA/Protein Isolation Procedure

DNA/RNA/protein isolation was applied using an Allprep DNA/RNA/Protein Mini Kit (Qiagen Inc.). Nanodrop measurements and 1.5% agarose gel electrophoresis were performed to check the quality and quantity of the isolated DNA and/or RNA.

### 2.5. Bisulfite Modification of DNAs

Bisulfite conversion is a method that makes changes in the chemical and physical characteristics of DNA. During bisulfite conversion, all nonmethylated cytosines are converted to uracil, and methylated cytosines are not converted [21]. All uracils (U) were changed to thymines (T) during PCR. Two hundred nanograms of DNA were denatured with heat, followed by a CT conversion reaction. After the stages of conversion, desulphonation and clean-up were completed, and the bisulfite modified DNA was obtained with EZ DNA Methylation-Gold Kit (Zymo RESEARCH) [22].

### 2.6. Real-Time Polymerase Chain Reaction

After the bisulfite modification process, DNA levels were measured with a spectrophotometer. Total 100 ng modified DNA was used for experiments. Methylation specific primers were designed with Methprimer software (The Li Lab, Department of Urology, UCSF). Methylated and unmethylated specific primers and probes were designed for* SBDS (138bp) (NM_016038.2)*,* TMPRSS2 (189bp) (NM_001135099.1)*, and* PAX5 (230 bp) (NM_016734.2)*. The methylated primers used in the study are listed in Table 1; unmethylated primers are shown in Table 2. The* β-Actin (121 bp) (NM_001101.4)* primers are used as reference gene primers and given in Table 3. Real-time PCR was performed using a Lightcycler 480 II (Roche Applied Sciences, Mannheim, Germany) for measuring methylation levels of the genes. Methylation levels were measured in 3 repeated experiments for each gene. Methylation ratios were calculated with the obtained Ct values according to the formula 2^−^(∆∆Ct) x 100. Results were calculated on average for three replicates.

### 2.7. Preparation of Positive and Negative Controls

Methylated and unmethylated controls were used to calculate the percentage of methylation. Placental DNA, which is known to be definitely unmethylated, was directly used as a negative control. Placental DNA was also used for preparing the methylation positive control. For this purpose, DNA was fully methylated using SssI methylase.

### 2.8. Statistical Analysis

All methylation data were analyzed using Fisher's exact Chi-square test (Fisher's exact test) using SPSS v.17.0 software (IBM Corp) because of the small set of samples and the presence of two variables only.

## 3. Results

**Figure 1:**
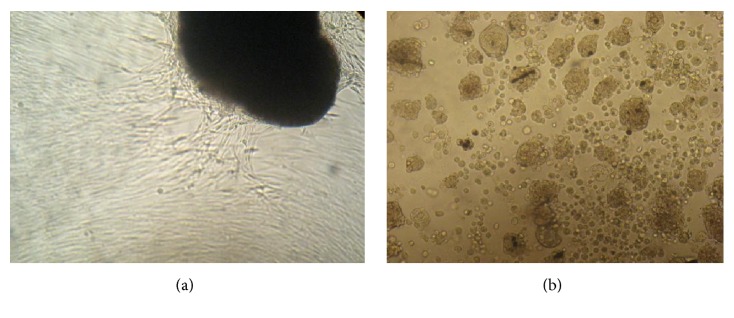
Illustrations of the cultures of primary tumor tissue and spheroid. (a) Primary cell cultures; (b) 3D spheroid cultures.

Primary tumor tissues from patients with breast cancer and malignant melanoma were cultivated in 2D and 3D culture systems (Figure 1). Cells were collected from primary tumors and cultivated tumor tissue samples. DNA materials were isolated from tumor tissues and the 2D and 3D culture cells of 10 patients with breast cancer (n=10) whose mean age was 56.5±12 years and 6 patients with malignant melanoma (n=6), mean age 49±14 years, were used for methylation analysis. The methylation levels of* PAX5, SBDS, and TMPRSS2* were examined according to ß-actin gene, which was the internal control gene.

### 3.1. Breast Cancer

**Figure 2:**
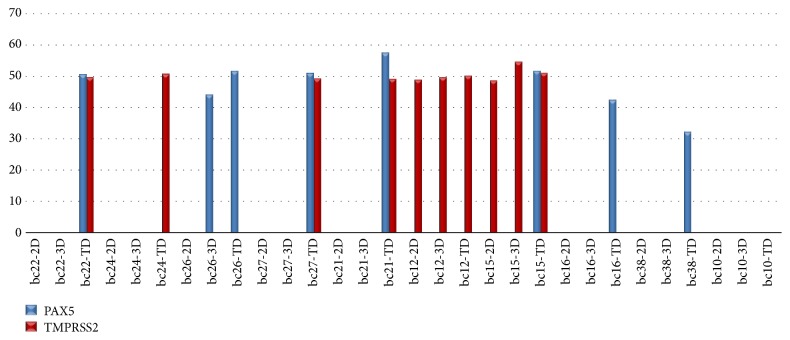
Methylation rates of patients with breast cancer: Figure shows methylation rates in patients with breast cancer. Methylation rates were found between 33 and 58% for* PAX5* gene and 49 and 55% for* TMPRSS2* gene, respectively. Four of 10 breast cancer primary tumor tissues were found as methylated for both* PAX5* and* TMPRSS2* (bc22, bc27, bc21, bc15). Methylation was not seen in 2D and 3D tissue culture systems for samples of bc22, bc24, bc27, bc21, bc16, bc38, bc10. Methylation in* PAX5* gene was not seen in any types of tissues of bc12 tumors. 2D: two-dimensional tissue culture, 3D: three-dimensional tissue culture, TD: primary tumor tissue, and BC: breast cancer.

The methylation rates in tissue DNA of patients with breast cancer were found between 33% and 58% for* PAX5* gene and between 49% and 55% for* TMPRSS2* gene according to* ß-actin *gene (Table 4) (Figure 2). Methylation on* SBDS* gene was not detected in all groups of patients with breast cancer (Table 5). The differences between cells grown in 2D-3D cultures and primary tumor tissues were statistically nonsignificant (P>0.05) for* SBDS* gene. In the patient group with breast cancer,* PAX5* was found methylated in 7 out of 10 (70%) tumor tissues, only one sample (10%) was observed as methylated in 3D culture, and no methylation was observed in any 2D culture samples (Table 5). There was no statistically significant difference in methylation levels between 2D and 3D culture systems (P>0.05); however, the difference both between 2D culture system and tumor tissues (P= 0.003) and between 3D culture system and tumor tissue was found statistically significant (P=0.02). There was no significant difference (P=0.17) between both methylation levels of 2D culture and tumor tissues and between 3D culture and tumor tissues for* TMPRSS2 *gene. Two samples (20%) were found methylated in 2D and 3D cultures and 6 samples were also observed as methylated (60%) in primary tumor tissue for* TMPRSS2* (P>0.05). The results were statically nonsignificant.* TMPRSS2 *could not be used as a marker for our* in vitro* model system because of differences of methylation levels in different types of tissue culture systems for breast cancer.

### 3.2. Malignant Melanoma

**Figure 3:**
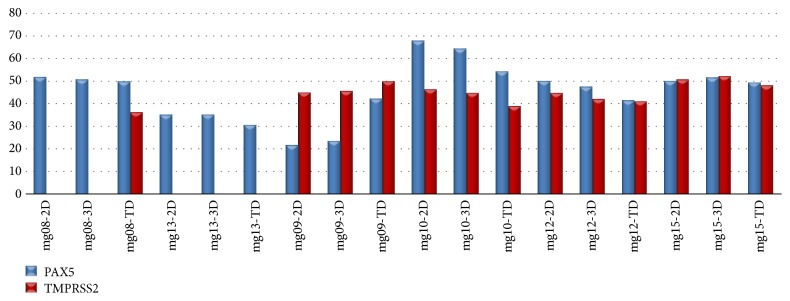
Methylation rates of malignant melanoma patients: methylation rates of* PAX5* gene were observed between 22 and 68% and 37 and 51% for* TMPRSS2* gene, respectively. Methylation in* PAX5* gene was seen in all malignant melanoma primary tissue and also grew tumor cells in 2D and 3D tissue culture systems (mg08-mg015). Two of 6 patients with malignant melanoma in 2D and 3D tissue culture systems did not show methylation for* TMPRSS2* gene (mg08 and mg013). 2D: two-dimensional tissue culture, 3D: three-dimensional tissue culture, TD: primary tumor tissue, and mg: malignant melanoma.

Methylation rates were observed between 22 and 68% for* PAX5* gene and between 37 and 51% of patients for* TMPRSS2* gene in tissue of patients with malignant melanoma according to* ß-actin* gene (Figure 3) (Table 6).* PAX5* gene was found methylated in all patients with malignant melanoma [6/6, (100%)] for primary tumor tissues and each type of culture (Table 5).* TMPRSS2* was observed as methylated in 2D and 3D culture in 4 samples [4/6, (67%)] and in tumor tissue samples of 5 patients [5/6, (83%)] (Table 5). At the* SBDS* gene, methylation was detected in a single tumor tissue sample and was determined as 17% (Table 5). There was no methylation detected in the 2D and 3D culture systems for* SBDS*. When all 3 genes were considered together in the analyses, there was no statistically significant difference in the methylation levels of all culture methods (P>0.05).

## 4. Discussion 

The main purpose of the study was to develop a biomarker to measure the* in vitro* effects of methylation inhibitors. For this purpose, our first aim was to cultivate the tumor cells in 2D and 3D tissue culture systems and to determine whether the primary tumor tissue and cultured cells in 2D and 3D tissue culture systems had the same methylation signature for* PAX5*,* TMPRSS2,* and* SBDS*. For this reason, appropriated primers and fluorescently labeled TaqMan probes were designed and methylation levels were investigated using real-time PCR. Upon the presence of ß-actin control gene, Ct values were obtained with real-time PCR, and methylation differences in the genes in question were calculated.

According to the results, it was shown that* SBDS *could be used as a negative control in* in vitro* model systems because the gene was completely unmethylated in primary breast tumor tissues and cultured breast tumor cells in 2D/3D culture systems. In our study groups, we found that methylation rates of primary breast cancer tissues were 70% (7/10) for* PAX5 *and 60% (6/10) for* TMPRSS2*, respectively. It was shown that* PAX5 *and* TMPRSS2 *were not suitable genes to measure the effects of methylation inhibitors in breast cancer tissue. Briefly, in this part of the study, we found that there was a requirement for new methylated genes other than* PAX5* and* TMPRSS2* to measure the effects of methylation inhibitor in breast cancer tissue in* in vitro* models.

In our study,* PAX5* gene was found methylated in all patients with malignant melanoma for primary tumor tissue and 2D/3D tissue culture systems. Therefore,* PAX5* gene was concluded to be the most appropriate gene that could be used to measure the effects of methylation inhibitors in* in vitro* models for malignant melanoma. Methylation of* TMPRSS2* gene was observed in some cases of malignant melanoma, but not in all. Thus, this gene was not an appropriate gene for use in* in vitro* systems. In addition, it was revealed that* SBDS* gene could not be used for the measurement of effects in methylation inhibitors in* in vitro* models because the methylation of* SBDS* was only detected in a single primary tumor tissue and was not observed in cells grown in 2D and 3D tissue culture systems. Methylation was observed in all investigated genes in other studies performed on cell lines; however, no methylation was observed in* SBDS* gene in our study. This situation suggests that cell lines may not reflect the real characteristics of primary tumor tissues for methylation.

In our study,* PAX5* gene was found methylated in primary malignant melanoma tumor tissues for the first time.* PAX5 *gene could also be used as a potential biomarker in our* in vitro* tissue culture systems to measure the effects of methylation inhibitors when malignant melanoma tissues are treated with methylation inhibitors such as 5-azacytidine and 5-azadeoxycytidine. The* in vitro* tissue culture systems presented in this article could be used for measuring the effects of methylation inhibitors and these systems could be used as* in vitro* diagnostic and prognostic tests to understand the effects of methylation inhibitors before giving them to patients with solid tumors. They could also be used to investigate the biologic effects of genes that change the methylation status with methylation inhibitors in different biologic pathways. The effects of methylation inhibitors such as 5-azacytidine and 5-azadeoxycytidine could be calculated by comparing the patient's clinical parameters and gene expression levels in different pathways, and the effectiveness of the drugs could be estimated for survival rates.

This was a preliminary study to understand whether solid tumor tissues were being grown in 2D and 3D culture environment. We also investigated whether* PAX5, SBDS, and TMPRSS2 *could be used as biomarkers by evaluating the stability of primary tumor cells grown in 2D or 3D culture media. For these purposes, 16 patients' materials for two tumor types and 3 different genes were chosen to test the feasibility of the method investigated in the study. We are planning to examine effects of 5-azacytidine (5-Aza C) in further research using genetic markers found here in this study.

In the future, we are planning to use* PAX5* gene as a biomarker for in vitro 2D/3D culture systems to examine the role of* PAX5* gene and/or other genes in the pathogenesis and biologic pathways of malignant melanoma in larger patient groups. We will also continue to research new biomarkers for breast cancer tissues to measure the effects of methylation inhibitors for* in vitro* systems.

## 5. Conclusion

Our results show that* PAX5* gene is an efficient marker to measure the effects of methylation inhibitors such as 5-azacytidine and 5-azadeoxycytidine at* in vitro* systems for malignant melanoma because the methylation levels are all the same in primary tissue and culture systems. Also, the results show that* SBDS* gene can be used as a negative control at* in vitro* model systems, since the gene was completely unmethylated in primary tumor tissue of breast and cultured breast tumor cells. The finding suggests that* SBDS* could be a suitable gene for the application of DNA demethylation agents.

Further research is needed for effective and high throughput analysis for detecting other genes and biomarkers for other types of cancer to measure the effects of methylation inhibitors for* in vitro* systems.

## Figures and Tables

**Table 1:** Methylated primers and probes.

***GENES ***	***SBDS (138bp)***	***TMPRSS2 (189bp)***	***PAX5 (230bp)***
FORWARD PRIMERS	5′-TGTAGTAGGCGATT	5′- AG GGA TTT TGG GGC	5′ AAA GCG TTC GAA
	TCGAAGCGT 3′	GTT TTT CG -3′	GGT ATC GTG A -3′

PROBES	6-FAM-GGGGGTGAAGAT	6-FAM-TCGAGGGTTCGA	6-FAM-AGC GCG TCG
	CGATATCGCGGTT-	GTTTGGGTTAAGGA -	TTT TAT ATT GTC GGT
	TAMRA	TAMRA	TCG G - TAMRA

REVERSE PRIMERS	5′- TCA ACC CTA ATT	5′- AAA ACG AAA CCG	5′TAC TAC TCT CCC GAC
	CGC CGA CTT CT -3′	ACC GCC TA -3′	TTC CCG-3′

**Table 2:** Unmethylated primers and probes.

***GENES***	***SBDS (138bp)***	***TMPRSS2 (189bp)***	***PAX5 (230bp)***
FORWARD PRIMERS	5′ TTT GTA GTA GGT	5′- ATA AGG GAT TTT	5′ AGT GTT TGA AGG
	GAT TTT GAA GTG 3′	GGG GTG TTT TTT G -3′	TAT TGT GAA ATG -3′

PROBES	6-FAM-TGG TGG GGG	6-FAM-TGT TTG AGG	6-FAM-AGT GTG TTG
	TGA AGA TTG ATA TTG	GTT TGA GTT TGG GTT	TTT TAT ATT GTT GGT
	TGG – TAMRA	AA - TAMRA	TTG GA - TAMRA

REVERSE PRIMERS	5′- AAT CAA CCC TAA	5′- AAA ACC AAA ACA	5′- TCT CCC AAC TTC
	TTC ACC AAC TT -3′	AAA CCA ACC ACC -3′	CCA CTC TA -3′

**Table 3:** Reference gene (*β*-actin) primers and probes.

**GENE **	**ACTB (121 bp)**
FORWARD PRIMER	5′-TGGTGATGGAGGAGGTTTAGTAAGT-3′

PROBE	VIC-ACCACCACCCAACACACAATAACAAACACA-TAMRA

REVERSE PRIMER	5′-AACCAATAAAACCTACTCCTCCCTTAA-3′

**Table 4:** Methylation rates (%) of three genes in patients with breast cancer (2D: two-dimensional tissue culture, 3D: three-dimensional tissue culture, T: primary tumor tissue, and BC: breast cancer).

**Specimens **		**Methylation Rates (**%**) **		
**Specimen 1 **		**bc22 - 2D **	**bc22 - 3D **	**bc22 – T **

**Genes **	***PAX5***	0	0	51
	***TMPRSS2***	0	0	50
	***SBDS***	0	0	0

**Specimen 2**		**bc24 - 2D **	**bc24 - 3D **	**bc24 – T **

**Genes **	***PAX5***	0	0	0
	***TMPRSS2***	0	0	51
	***SBDS***	0	0	0

**Specimen 3**		**bc26 - 2D **	**bc26 - 3D **	**bc26 – T **

**Genes **	***PAX5***	0	44	52
	***TMPRSS2***	0	0	0
	***SBDS***	0	0	0

**Specimen 4**		**bc27 - 2D **	**bc27 - 3D **	**bc27 – T **

**Genes **	***PAX5***	0	0	51
	***TMPRSS2***	0	0	49
	***SBDS***	0	0	0

**Specimen 5**		**bc21 - 2D **	**bc21 - 3D **	**bc21 – T **

**Genes **	***PAX5***	0	0	58
	***TMPRSS2***	0	0	49
	***SBDS***	0	0	0

**Specimen 6**		**bc12 - 2D **	**bc12 - 3D **	**bc12 – T **

**Genes **	***PAX5***	0	0	0
	***TMPRSS2***	49	50	50
	***SBDS***	0	0	0

**Specimen 7**		**bc15 - 2D **	**bc15 - 3D **	**bc15 – T **

***Genes***	***PAX5***	0	0	52
	***TMPRSS2***	49	55	51
	***SBDS***	0	0	0

**Specimen 8**		**bc16 - 2D **	**bc16 - 3D **	**bc16 – T **

**Genes **	***PAX5***	0	0	43
	***TMPRSS2***	0	0	0
	***SBDS***	0	0	0

**Specimen 9**		**bc38 - 2D **	**bc38 - 3D **	**bc38 – T **

**Genes **	***PAX5***	0	0	33
	***TMPRSS2***	0	0	0
	***SBDS***	0	0	0

**Specimen 10**		**bc10 - 2D **	**bc10 - 3D **	**bc10 - T **

**Genes **	***PAX5***	0	0	0
	***TMPRSS2***	0	0	0
	***SBDS***	0	0	0

**Table 5:** Methylation status of genes according to groups of patients and cultivation methods (2D: two-dimensional tissue culture, 3D: three-dimensional tissue culture, and T: primary tumor tissue).

**Genes **	**Patients **	**Cell culture method **	**Total number ** **of patients **	**Absence of Methylation **		**Presence of Methylation **	
				**Number of patients (n) **	%	**Number of patients (n) **	%
***PAX5***	Breast ca	2D	10	10	100	0	0

		3D	10	9	90	1	10

		Primary tumor tissue	10	3	30	7	70

	Melanoma	2D	6	0	0	6	100

		3D	6	0	0	6	100

		Primary tumor tissue	6	0	0	6	100

***TMPRSS2***	Breast ca	2D	10	8	80	2	20

		3D	10	8	80	2	20

		Primary tumor tissue	10	4	40	6	60

	Melanoma	2D	6	2	33	4	67

		3D	6	2	33	4	67

		Primary tumor tissue	6	1	17	5	83

***SBDS***	Breast ca	2D	10	10	100	0	0

		3D	10	10	100	0	0

		Primary tumor	10	10	100	0	0

		tissue					

	Melanoma	2D	6	6	100	0	0

		3D	6	6	100	0	0

		Primary tumor tissue	6	5	83	1	17

**Table 6:** Methylation rates of patients with malignant melanoma (2D: two-dimensional tissue culture, 3D: three-dimensional tissue culture, T: primary tumor tissue, and mg: malignant melanoma).

**Specimens **	**Methylation Rates (**%**) in **		
	**2 Dimensional Cell Culture (**%**) **	**3 Dimensional Cell Culture (**%**) **	**Primer Tumor Tissue (**%**) **

**Specimen 1 **			

***PAX5***	**52 **	**51 **	**50 **

***TMPRSS2***	**0 **	**0 **	**37 **

***SBDS***	**0 **	**0 **	**0 **

**Specimen 2 **			

***PAX5***	**35 **	**35 **	**31 **

***TMPRSS2***	**0 **	**0 **	**0 **

***SBDS***	**0 **	**0 **	**0 **

**Specimen 3 **			

***PAX5***	**22 **	**24 **	**42 **

***TMPRSS2***	**45 **	**46 **	**50 **

***SBDS***	**0 **	**0 **	**38 **

**Specimen 4 **			

***PAX5***	**68 **	**65 **	**55 **

***TMPRSS2***	**47 **	**45 **	**39 **

***SBDS***	**0 **	**0 **	**0 **

**Specimen 5 **			

***PAX5***	**50 **	**48 **	**42 **

***TMPRSS2***	**45 **	**42 **	**41 **

***SBDS***	**0 **	**0 **	**0 **

**Specimen 6 **			

***PAX5***	**50 **	**52 **	**50 **

***TMPRSS2***	**51 **	**52 **	**48 **

***SBDS***	**0 **	**0 **	**0 **

## Data Availability

The data used to support the findings of this study are included within the article.

## Abbreviations

2D: Two-dimensional
3D: Three-dimensional.

## References

[1] Ferlay J., Shin H. R., Bray F., Forman D., Mathers C., Parkin D. M. (2010). Estimates of worldwide burden of cancer in 2008: GLOBOCAN 2008. *International Journal of Cancer*.

[2] Nikolaou V., Stratigos A. J. (2014). Emerging trends in the epidemiology of melanoma. *British Journal of Dermatology*.

[3] Lauss M., Ringnér M., Karlsson A. (2015). DNA methylation subgroups in melanoma are associated with proliferative and immunological processes. *BMC Medical Genomics*.

[4] Fu S., Wu H., Zhang H., Lian C. G., Lu Q. (2017). DNA methylation/hydroxymethylation in melanoma. *Oncotarget *.

[5] Moran B., Silva R., Perry A. S., Gallagher W. M. (2018). Epigenetics of malignant melanoma. *Seminars in Cancer Biology*.

[6] de Unamuno Bustos B., Murria Estal R., Pérez Simó G. (2018). Aberrant DNA methylation is associated with aggressive clinicopathological features and poor survival in cutaneous melanoma. *British Journal of Dermatology*.

[7] Pirola L., Ciesielski O., Balcerczyk A. (2018). The methylation status of the epigenome: its emerging role in the regulation of tumor angiogenesis and tumor growth, and potential for drug targeting. *Cancers*.

[8] Rahman N. (2014). Realizing the promise of cancer predisposition genes. *Nature*.

[9] Polyak K. (2007). Breast cancer: Origins and evolution. *The Journal of Clinical Investigation*.

[10] Li S. Y., Wu H. C., Mai H. F. (2018). Microarray-based analysis of whole-genome DNA methylation profiling in early detection of breast cancer. *Journal of Cellular Biochemistry*.

[11] Rothhammer T., Bosserhoff A.-K. (2007). Epigenetic events in malignant melanoma. *Pigment Cell Research*.

[12] Ehrich M., Turner J., Gibbs P. (2008). Cytosine methylation profiling of cancer cell lines. *Proceedings of the National Acadamy of Sciences of the United States of America*.

[13] Nutt S. L., Heavey B., Rolink A. G., Busslinger M. (1999). Commitment to the B-lymphoid lineage depends on the transcription factor Pax5. *Nature*.

[14] Desouki M. M., Post G. R., Cherry D., Lazarchick J. (2010). PAX-5: a valuable immunohistochemical marker in the differential diagnosis of lymphoid neoplasms. *Clinical Medicine & Research*.

[15] Lin B., Ferguson C., White J. T. (1999). Prostate-localized and androgen-regulated expression of the membrane-bound serine protease TMPRSS2. *Cancer Research*.

[16] Donaldson S. H., Hirsh A., Li D. C. (2002). Regulation of the epithelial sodium channel by serine proteases in human airways. *The Journal of Biological Chemistry*.

[17] Ganapathi K. A., Austin K. M., Lee C.-S. (2007). The human Shwachman-Diamond syndrome protein, SBDS, associates with ribosomal RNA. *Blood*.

[18] Shammas C., Menne T. F., Hilcenko C. (2005). Structural and mutational analysis of the SBDS protein family: Insight into the leukemia-associated shwachman-diamond syndrome. *The Journal of Biological Chemistry*.

[19] Xu P., Hu G., Luo C., Liang Z. (2016). DNA methyltransferase inhibitors: an updated patent review (2012-2015). *Expert Opinion on Therapeutic Patents*.

[20] Harman R. M., Curtis T. M., Argyle D. J., Coonrod S. A., Van de Walle G. R. (2016). A comparative study on the in vitro effects of the DNA methyltransferase inhibitor 5-Azacytidine (5-AzaC) in breast/mammary cancer of different mammalian species. *Journal of Mammary Gland Biology and Neoplasia*.

[21] Grunau C., Clark S. J., Rosenthal A. (2001). Bisulfite genomic sequencing: systematic investigation of critical experimental parameters. *Nucleic Acids Research*.

[22] Ehrich M., Zoll S., Sur S., van den Boom D. (2007). A new method for accurate assessment of DNA quality after bisulfite treatment. *Nucleic Acids Research*.

